# A simplicial analysis of the fMRI data from human brain dynamics under functional cognitive tasks

**DOI:** 10.3389/fnetp.2022.924446

**Published:** 2022-08-23

**Authors:** Rabindev Bishal, Sarika Cherodath, Nandini Chatterjee Singh, Neelima Gupte

**Affiliations:** ^1^ Department of Physics, Indian Institute of Technology Madras, Chennai, India; ^2^ National Center for Brain Research, Gurgaon, India

**Keywords:** fMRI, brain networks, simpicial characterisers, time series, reading tasks

## Abstract

The topological analysis of fMRI time series data has recently been used to characterize the identification of patterns of brain activity seen during specific tasks carried out under experimentally controlled conditions. This study uses the methods of algebraic topology to characterize time series networks constructed from fMRI data measured for adult and children populations carrying out differentiated reading tasks. Our pilot study shows that our methods turn out to be capable of identifying distinct differences between the activity of adult and children populations carrying out identical reading tasks. We also see differences between activity patterns seen when subjects recognize word and nonword patterns. The results generalize across different populations, different languages and different active and inactive brain regions.

## 1 Introduction

Functional magnetic resonance imaging (fMRI) measurements provide patterns of brain activity during specific tasks with respect to control conditions. To distinguish if patterns of brain activity might characterize specific brain states, recent research has sought to use simplicial analysis to identify metric spaces that optimally distinguish brain states across experimentally defined conditions (Bashan et al., 2012; Bartsch et al., 2015; Simas et al., 2015; Bianconi, 2021; Jacob et al., 2021).

The purpose of the present study is to explore the identification of metric spaces to characterize reading populations. The fMRI data obtained from children and adults reading words and nonwords, matched in performance, showed common regions of activation across both populations. The time series obtained from common regions of activation from both populations was used to construct a complex network (a time series network (Zhang and Small, 2006)), further subjected to simplicial analysis using methods of algebraic topology (Jonsson, 2008). This analysis can provide insights into the short term correlations in the time series in different regions of imaging in the brain, as well as different segments of the reading task and distinct reading populations. We hypothesize that a comparison of the topology of reading networks (Cherodath and Singh, 2015) might provide novel insights into reading patterns in adult and child populations.

We analyze fMRI data obtained from subjects carrying out reading tasks in two languages, English and Hindi. The fMRI paradigms employed involved reading tasks in Hindi and English in a simple and identical block design. The task comprised of alternating word and nonwords reading blocks separated by rest blocks with a visual baseline. Participants were instructed to read aloud words and nonwords that appeared during the task blocks, and to fixate on the symbol strings displayed during rest blocks without any oral response. In each task block either 10 words or nonwords were presented, each item appearing for a duration of 2 s at the center of the screen. During the length of the task in each language, 60 words and 60 nonwords were presented, with each item randomly selected from a word or nonword list respectively. During every rest block, one false font string from a list of four false font stimuli was randomly selected and presented for 20 s. The participants performed two runs of the fMRI task in each language, where each run consisted of 3 word blocks, 3 nonword blocks and 6 rest blocks. The total duration of the reading task was 16 min. The details of the stimuli used are presented in Ref. (Cherodath and Singh, 2015). The fMRI task performance accuracies of adult and children groups, calculated from in-scanner vocal responses during various tasks conditions were compared. Although adults showed trends of higher accuracies than children, the group differences were not statistically significant in any condition. Therefore, the two groups were matched on task performance, ruling out performance effects on activation patterns.The neuroimaging results were subjected to the following statistical analysis. Two sample t tests were performed to explore group differences in neural activity in the combined contrast of all reading conditions versus baseline (FDR *p* < 0.05, *k* > 10). Results revealed higher levels of activity in the right hippocampus in children. On the other hand, adults showed higher activity in a network of regions - bilateral articulatory motor regions, putamen and thalamus, right superior temporal region, supramarginal gyrus, inferior frontal region and the left fusiform gyrus.

The fMRI data from the Lputamen and RSTG (Right Superior Temporal Gyrus) which are activated in both children and adults are subjected to simplicial analysis. A comparison of the characteristics observed for both cases is carried out. Our pilot study reveals differences between the networks for adults and children, across differentiated reading tasks, and across different languages. The differences are seen more strongly in active regions. We also carry out a comparison of activity in highly active and less active regions during the task, as well as in the recognition of words and nonwords. The terms “active” and “less active” have been quantified in terms of the BOLD (blood oxygen level-dependent) signal seen in the fMRI images in Ref (Cherodath and Singh, 2015). This is a measure associated with blood flow and blood oxygenation levels in the regions in the brain which is known to be correlated with neuronal activity.” Further information on the BOLD signal can be found at https://www.ncbi.nlm.nih.gov/pmc/articles/PMC6859204.

## 2 The TS network and simplicial analysis

**FIGURE 1:**
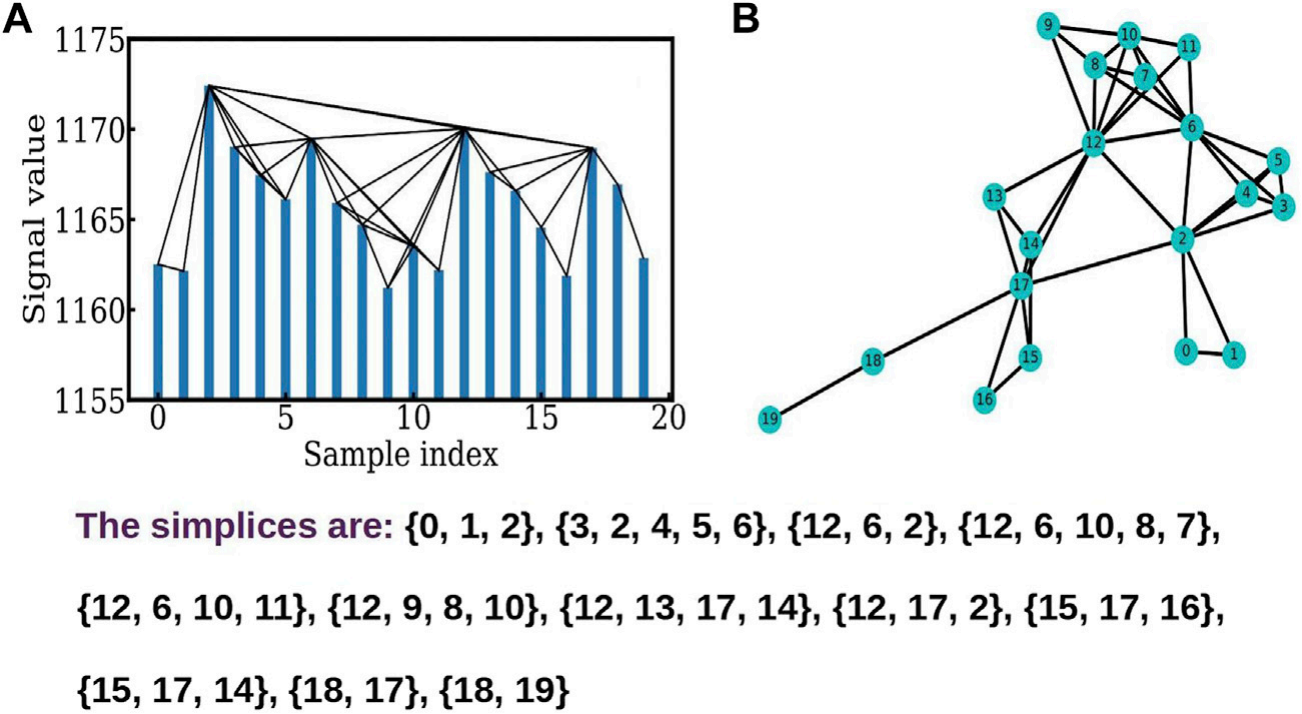
**(A)** shows the connections between the nodes obtained using the visibility algorithm. **(B)** The corresponding constructed network showing different simplices.

The time series described here are the time series from fMRI data obtained in the case of sets of 5 adults and 5 children carrying out a reading task, and are averaged over all the members of each set. The time series consists of 240 data points sampled at an interval of 3 s. The highly active regions picked up here for analysis were the Lputamen and RSTG and two less active regions viz. L1 (Lingual Gyrus) and S1 (a prefrontal region). We present here the results of the analysis of the Lputamen and L1 regions. The results for the RSTG and S1 are similar, and hence are not shown here. The graphs can be downloaded from the site https://github.com/tsardb/f_vector_results. The time series network is constructed here using the visibility algorithm (Lacasa et al., 2008) which is outlined in Figure 1. The visibility algorithm is implemented by connecting two points (*y*_*i*_, *t*_*i*_) and (*y*_*j*_, *t*_*j*_) by a straight line, provided no other intermediate point, (*y*_*r*_, *t*_*r*_) lies above the line, i.e. (*y*_*i*_, *t*_*i*_) and (*y*_*j*_, *t*_*j*_) should be “visible” to each other, with no other intermediate point obstructing the line of visibility in between (Figure 1A). For this, all intermediate points should satisfy the condition

$$y_{j}>y_{r}+\frac{y_{j}-y_{i}}{t_{j}-t_{i}}\left(t_{j}-t_{r}\right)$$ (1)

Figure 1A shows the visibility algorithm (Lacasa et al., 2008) for the time series of the fMRI data used here. The time series is sampled at intervals of, up to the first points, and the neighbors of each node, i.e. the points which are visible to each other, are seen in the graph. The network graph, and its connectivity matrix is constructed by setting up a link between all pairs *i* and *j* which are visible to each other using the algorithm above. The network graph so constructed is shown in Figure 1B. The simplicial structures are identified using the Bron-Kerbosch algorithm (Bron and Kerbosch, 1973). The graph shows simplicial structures (Bron and Kerbosch, 1973; Johnston, 1976) of dimension ranging from 1 to 4.

The network graphs so generated are then analyzed using recently constructed topological characterisers or simplicial characterisers. Such characterisers have been known to yield useful information in the analysis of neural data (Sizemore et al., 2019) as well as chaotic systems (Chutani et al., 2020), traffic on networks (Andjelković et al., 2015a), social networks (Andjelković et al., 2015b) etc. The specific characteriser used here is the quantity called the *f* − vector which has proved to be of utility earlier in the analysis of neural data (Giusti et al., 2016). We define this quantity in the next subsection.

### 2.1 The definition of the *f* − vector

The *f* − vector analysis for a time series network, is a useful handle on the short term correlations in the system. The TS network is constructed using the visibility algorithm (Lacasa et al., 2008) and is analyzed by identifying the cliques (Bron and Kerbosch, 1973; Johnston, 1976) and simplicial complexes. Here, cliques are complete sub-graphs, and simplices are sets of connected nodes, with isolated nodes, two nodes connected by a link, and three nodes connected by links being examples of 0, 1, and 2 dimensional simplices (Andjelković et al., 2015a; Andjelković et al., 2015b; Chutani et al., 2020). It is clear from the Figure 1B that the simplicial structure reflects the short term correlations in the system. Figure 1B shows the triangles and tetrahedra present in this part of the data. The number of *q* − dimensional simplices, forms the *q* − th component of the *f* − vector, where the $\vec{f}=\left\{f_{0},f_{1},\ldots ,f_{q_{\max}}\right\}$ , and *q*_max_ is the dimension of the highest dimensional simplices in the network. We note that *n* − point correlations in the data will lead to simplicial complexes of higher dimensions, and higher values of *q*_max_. The statement that *n* − point correlations in the data will lead to simplicial complexes of higher dimensions, and higher values of *q*_max_ can be motivated in terms of Figures 1A,B. If a time series value *x*_1_ is visible to its neighbours *x*_0_ and *x*_2_ it is a part of the simplex or clique 0, 1, 2 (Figure 1B). The value *x*_2_ is further visible to the variables *x*_3_, *x*_4_, *x*_5_ and *x*_6_, which form a clique again. Thus the variable *x*_2_ can be said to be a part of the correlation $<x_{2}x_{3}x_{4}x_{5}x_{6}>$ as well as higher ones. Since the variable labels here are temporal labels in the time series, these are reflections of temporal correlations in the system. These are short term correlations, as variables which are close to each other in time are likely to be visible to each other, and hence will be a part of the same simplicial complex whose dimension depends on the number of variables involved. Thus higher order correlations lead to simplicial complexes of higher dimensions, and higher values of *q*_max_.

## 3 The *f* − vectors for the *f*MRI data

The *f* − vectors of the system contain information about the short term correlations of the system, and reflect the correlations of the system in terms of the number of simplices of each type, and the complexity of the structures. The nature of the time series and the regions of imaging have been described above. We note that many pairwise comparisons are possible, e.g. between words, nonwords and rests, between different languages (here, English and Hindi), between active and inactive regions, and many other combinations. We looked for combinations where the differences were clear, and were also robust to changes in the details. Within our observations, the clearest differences were as follows:

### 3.1 Comparison between adults and children

We show below the *f* − vector comparison between English words and nonwords in English for an active region, in this case the Lputamen. We consider adults and children separately.

**FIGURE 2:**
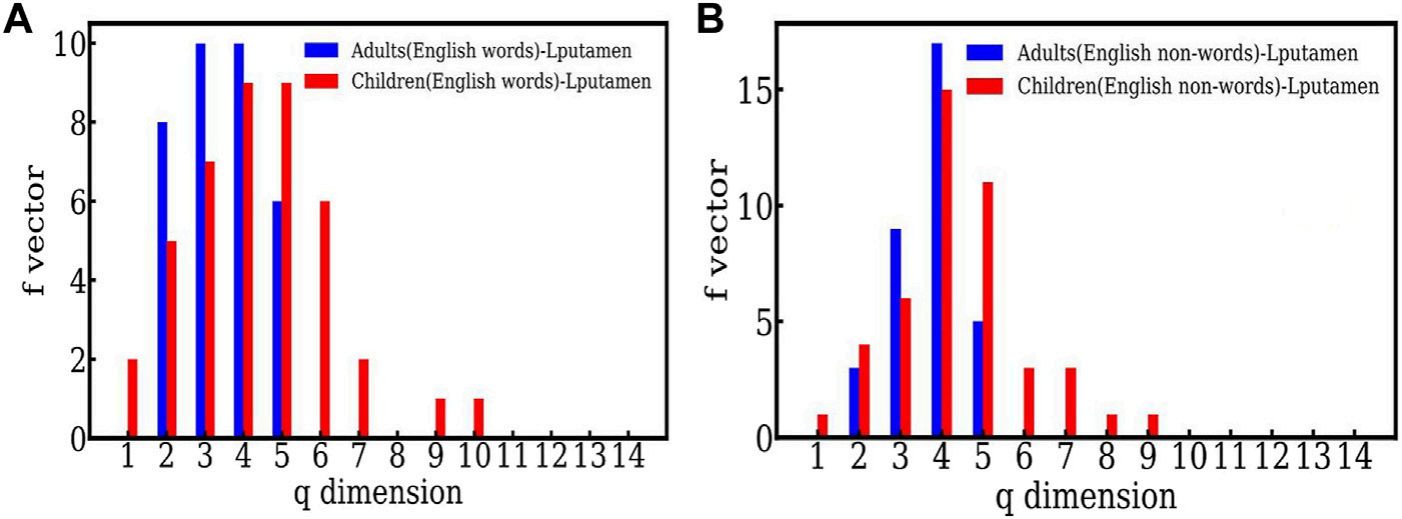
**(A)** The comparison between the f-vector of an active (Lputamen) region for adults and children in the task of reading English words. **(B)** The comparison between the f-vector of an active (Lputamen) region for adults and children in the task of reading English nonwords.

The *f* − vector distributions seen in Figure 2 for English words and English nonwords, both clearly indicate that the simplices seen in the case of children are higher dimensional as compared to adults. For e.g. Figure 2A shows that in the case of words, the highest dimensional simplex seen for adults is 5 dimensional, whereas the highest dimension seen in the case of children is 10. Similarly for English nonwords (Figure 2B), the highest dimension seen is 5 for adults and 9 for children. We suggest that this indicate that adults are able to resolve the words and nonwords within 5 − point correlations, and hence within shorter time scales, as explained above. On the other hand, the time series for children show higher order structures, which reflects higher correlations. Thus children need to correlate more data, and need more time, to resolve the same word and nonword information than the adults. Hence we suggest that the adults process the information more efficiently. We also note that this behavior was seen for both English and Hindi, and also for another active sampling region (viz. LSG).

### 3.2 Comparison between words and nonwords: English

**FIGURE 3:**
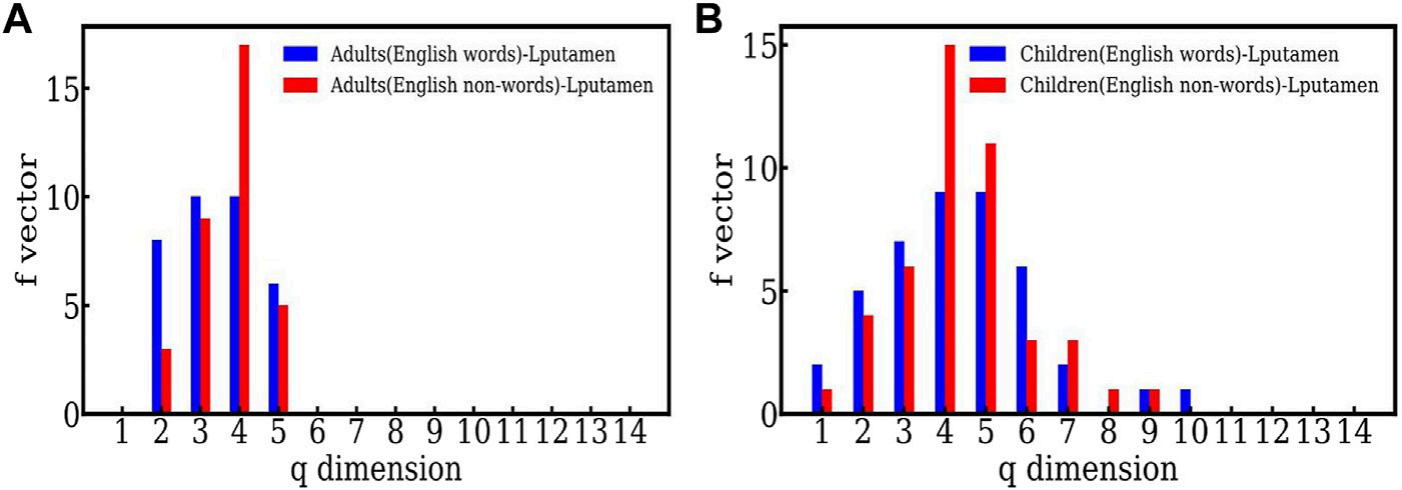
**(A)** The *f* − vector of the simplicial complex of the active (Lputamen) region for adults carrying out a reading task involving English words and nonwords. **(B)** The *f* − vector of the simplicial complex of the active (Lputamen) region for the reading task with English words and nonwords in the case of children.

Figure 3 plots the same data for the Lputamen in another way, namely the two populations, adults and children for words (Figure 3A) and nonwords (Figure 3B). Here, the number of simplices for words is about the same for both groups. However, in the case of nonwords, the number of simplices of dimension 5 is much larger in children. This suggests that the efficiency of the networks in children is similar to that in adults when reading English words, but not English nonwords. The higher order correlations seen in children for English nonword reading may be indicative of their lower efficiency owing to the relatively high difficulty of reading nonwords as compared to words. Overall, the findings suggest adult-like efficiency in reading English words, but not the more difficult stimuli—English nonwords.

We note that the largest number of simplices have dimension 4 here, for both words and nonwords. This appears at the dimension 3 in the case of Hindi. We speculate that this may be some indicator of the language structure. Further studies are required to make a statement on this.

### 3.3 The comparison between words and nonwords: Hindi

**FIGURE 4:**
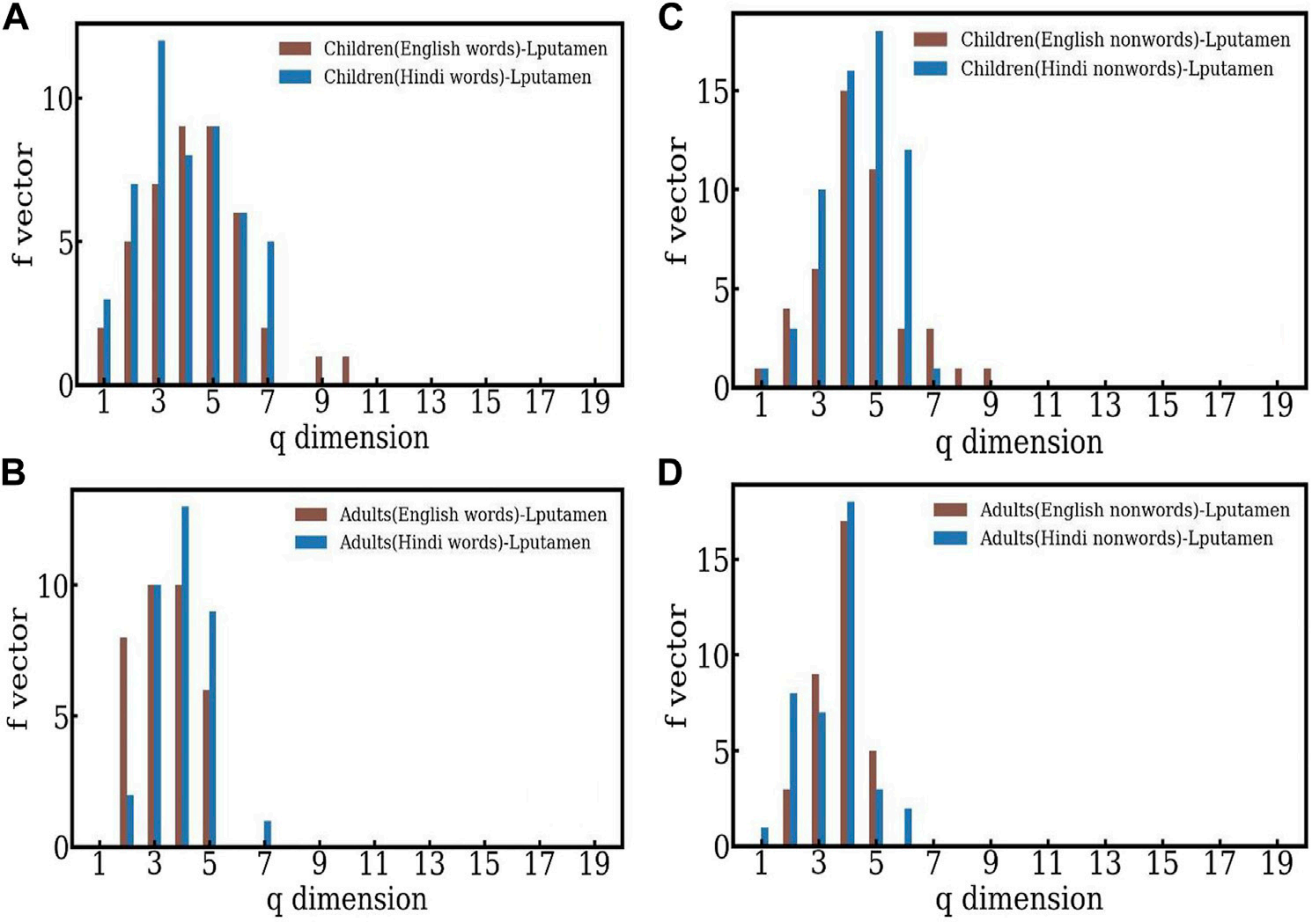
**(A)**–**(B)** The comparison of the f-vectors computed for children and adults in the case of English and Hindi words respectively. **(C)**–**(D)** The comparison of the f-vectors computed for children and adults in the case of English and Hindi nonwords respectively.

It is interesting to see if the language chosen for the reading task affects structure of the *f* − vector distribution. To see this, we study the *f* − vector distributions for Hindi words and nonwords, and compare them with the behavior seen for English words and nonwords (see Figure 4). Here, again the structures are skewed towards to lower *q* − levels, indicating more efficient separation between words and nonwords in the case of adults. In the case of children, the word-nonword distinction is less efficient in Hindi as well. Thus both conclusions are in agreement with those seen in the case of English, indicating that the adults process the distinction between word-nonword information more efficiently. We note that the structures seen in the case of children have lower dimensions than those seen for English, both for words and nonwords.

### 3.4 Comparison between regions of high and low activity

We now carry out a comparison of a region of high (Lputamen) and low (L1) activity, as indicated by the highlights in the fMRI in Ref. (Cherodath and Singh, 2015). The high and low activity is as measured in terms of the BOLD signal described above. We have considered the English reading task, which consists of 240 data points. There are three cycles in the reading tasks. Each cycle contains 80 points which follow the sequence words, rest, nonwords, rest, respectively; each section contains twenty points. Thus the time series samples all the elements of the reading task over the two regions.

**FIGURE 5:**
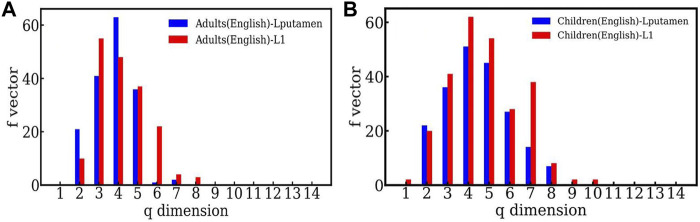
**(A)** The *f*-vector of the simplicial complex of one active (Lputamen) and one inactive (L1) region in the case of the English language reading task respectively. Both the cases are for the adults. **(B)** The *f* − vector of the high activity (Lputamen) region and the low activity (L1) region for the English reading task, in the case of children.

Figure 5A, shows the *f* − vector of the TS network constructed from time-series data of the high activity region (Lputamen) and low activity region (L1) in the case of the English reading task by adults. The highly active region has more simplices of lower dimensions than the region of less activity, indicating the dominant short range structure of the correlations. Figure 5B shows the comparison between the highly active and less active regions for children for the English reading task. Here again, higher dimensional structures are seen for both the highly active and less active regions, but the distinction between the two regions is less sharp than that seen for the adults. We note that the behavior seen in other choices of highly active and less active regions,viz. RSTG and S1 is similar. As mentioned above, the plots and further details can be found at https://github.com/tsardb/f_vector_results.

### 3.5 Usual network characterisers

We have carried out a comparison between various pairs of data here using usual network characterisers. It is also interesting to see if the usual network characterisers show any signatures of the behavior seen here. The usual characterisers seen here are listed in Table 1.

**TABLE 1:** The 2^*nd*^—5^*th*^ columns of the table correspond to the average degree (D_*avg*_), average clustering coefficient (C_*avg*_) average path length and diameter (L_*avg*_) of the TS network constructed for the time series for different tasks (English words and nonwords) in the Lputamen regions respectively.

Data set	D_*avg*_	C_*avg*_	L_*avg*_	Diameter
Adults-English-words	5.97	0.81	2.75	6
Adults-English-nonwords	6.33	0.8	2.62	5
Children-English-words	8.33	0.76	2.58	6
Children-English-nonwords	8.20	0.76	2.81	5
English reading tasks (Adults)	7.0	0.77	4.08	10
English reading tasks (Children)	8.83	0.76	3.58	8

The quantities studied here are the average degree, the average clustering co-efficient and the average shortest path length of the network. The average degrees (*D*_*avg*_) are smaller for adults than for children. The same effect is seen for the clustering coefficients and *L*_*avg*_, although the changes are small. For the full time series (last two lines), the results are unclear. Thus the usual clustering coefficients show no clear signature of the population, and the simplicial characterisers do a much better distinction.

## 4 Conclusion

We use the methods of algebraic topology to characterize the TS networks obtained from fMRI data via the visibility algorithm for adult and child populations carrying out reading tasks. Our methods look at the *f* − vector, which counts the number of simplices of each type, and thus is a measure of the complexity of short term correlations in the series. The adult populations consistently show lower dimensional simplicial structure than the child population across different segments of the reading task. This indicates the dominance of short term correlations in the adult populations, as compared to the child population, and can be indicative of the fact that the children take longer to process the reading information. Thus the adults demonstrate more efficient analysis, presumably due to their longer experience in such tasks. There is also a differentiation between the *f* − vector distributions seen for the word and nonword segments of the reading tasks, which indicates higher efficiency for the adults for both cases. Within each population, the word and nonword distributions show distinct behavior, for two different languages. However, the reading circuits in the brain for adults and children are different, so further analysis is required here. Finally, we note that similar effects are seen in the less active regions as well, indicating some response to the stimuli. However, the effects are more marked in the case of the highly active region.

To conclude, the simplicial characterization of TS networks constructed from brain data, can provide valuable information about the structure, function and hidden geometry of cognitive networks. This characterization can complement and support conclusions drawn from older methods of analysis. We note that other simplicial characterisers are also available which can further this analysis. We hope this pilot study will provoke further and more extensive studies in the field.

## Data Availability

The original contributions presented in the study are included in the article/Supplementary Materials, further inquiries can be directed to the corresponding author.
